# Cerebral Projection of Mirrored Touch via sLORETA Imaging

**DOI:** 10.3390/life13051201

**Published:** 2023-05-17

**Authors:** Dita Dubová, Dominika Dvořáčková, Dagmar Pavlů, David Pánek

**Affiliations:** Faculty of Physical Education and Sport, Charles University, 162 52 Prague, Czech Republic; ditadubova@gmail.com (D.D.); dvorackova.dominika@ftvs.cuni.cz (D.D.); panek@ftvs.cuni.cz (D.P.)

**Keywords:** haptic contact, touch, mirror neurons, mirror therapy, neuroplasticity, sensorimotor, Brodmann areas, EEG, sLORETA

## Abstract

Touch is one of the primary communication tools. Interestingly, the sensation of touch can also be experienced when observed in another person. Due to the system of mirror neurons, it is, in fact, being mapped on the somatosensory cortex of the observer. This phenomenon can be triggered not only by observing touch in another individual, but also by a mirror reflection of the contralateral limb. Our study aims to evaluate and localize changes in the intracerebral source activity via sLORETA imaging during the haptic stimulation of hands, while modifying this contact by a mirror illusion. A total of 10 healthy volunteers aged 23–42 years attended the experiment. The electrical brain activity was detected via scalp EEG. First, we registered the brain activity during resting state with open and with closed eyes, each for 5 min. Afterwards, the subjects were seated at a table with a mirror reflecting their left hand and occluding their right hand. The EEG was then recorded in 2 min sequencies during four modifications of the experiment (haptic contact on both hands, stimulation of the left hand only, right hand only and without any tactile stimuli). We randomized the order of the modifications for each participant. The obtained EEG data were converted into the sLORETA program and evaluated statistically at the significance level of *p* ≤ 0.05. The subjective experience of all the participants was registered using a survey. A statistically significant difference in source brain activity occurred during all four modifications of our experiment in the beta-2, beta-3 and delta frequency bands, resulting in the activation of 10 different Brodmann areas varying by modification. The results suggest that the summation of stimuli secured by interpersonal haptic contact modified by mirror illusion can activate the brain areas integrating motor, sensory and cognitive functions and further areas related to communication and understanding processes, including the mirror neuron system. We believe these findings may have potential for therapy.

## 1. Introduction

Touch is one of the primary communication tools amongst individuals. Because it bears emotional components, touch plays a crucial role in social interactions and expressions of empathy [1]. In healthcare, touch has many purposes including care, attention, as well as physical and emotional relief [2,3]. Physiotherapists use touch not only as a communication instrument, but also for diagnostical and therapeutical purposes [4].

Interestingly, the sensation of touch can also be experienced in cases when it is only observed in another person, without receiving the actual physical stimulus. This is possible thanks to the system of mirror neurons in the brain [5,6].

The mirror neuron system is one of the most important neuroscientific discoveries of the last several decades [7,8]. Mirror neurons are specific cells that fire during purpose-motivated movement execution, but also while such movement is solely observed in another individual [8,9,10]. 

These principles also take place in the sensorimotor system. Observing touch can activate relevant brain areas; therefore, the observed touch is in fact being mapped on the somatosensory cortex of the observer [5,6]. This system can help us anticipate the consequences of the observed touch on our own body, and to understand the effect of such stimulus experienced by someone else [5]. 

Mirror neurons can be also discharged by a mirror illusion [11].

This mechanism is commonly used in rehabilitation as the mirror visual feedback (MVF). One of the methods using the mechanism in practice is known simply as mirror therapy (MT). MT treatment aims to trigger the brain’s neuroplasticity which facilitates the growth of new neural synapses and enhances the existing ones. The ability of the central nervous system to renew neural connections is dependent on their training. It is enabled by activating mirror neurons in the contralateral brain hemisphere based on the feedback delivered by the visual illusion provided by a mirror [12,13,14,15]. 

Many studies have proven that mirror neurons and MVF can be successfully used in the rehabilitation of motor skills. The important advantage is that the training can take place even when the actual movement is impossible [7,14,16,17]. Sensory-wise, the MVF can positively affect conditions such as chronical pain, complex regional pain syndrome, phantom pains and more [7].

Available studies show that observing touch in others can activate somatosensory cortices S1 and S2 [1,6]. 

Nakanishi et al. (2018) conducted an experiment using magnetoencephalographic recordings of somatosensory-evoked field responses to tactile stimulation of the index finger on both hands in three tasks and one control task. In their study, they aimed to analyze the somatosensory cortex response to tactile stimulation with and without mirror illusion. In the experiment, the researchers included eight healthy right-handed participants. The offered stimulus was mechanical, provided by a custom-designed machine. Their measurements proved increased activity in the contralateral somatosensory cortex, specifically in the area representing the hand. Additionally, in six out of the eight participants, the changes also occurred in the secondary somatosensory area both ipsilateral and contralateral to the stimulated limb. There was a statistically significant difference between the mirror and non-mirror task, which proves that observing touch in a mirror activates contralateral somatosensory cortices [18]. 

Additionally, other brain areas respond to mirror illusion. Arya (2016) published a review of neural mechanisms of mirror therapy and described extensive brain activity in areas which cannot be achieved otherwise. The therapy increases activity in brain regions including the precuneus and the posterior cingulate cortex, areas within the occipital lobe, dorsal frontal area, corpus callosum and the premotor cortex, along with the primary motor and the primary somatosensory cortices [19]. 

From related studies, we know that touch enhances visual perception [5,20,21] and other modalities [22,23]. Finally, based on the gateway theory, touch has analgetic effects on both the spinal and supraspinal level [24].

However, there is not much data available specifying the precise localization of the changes in the central nervous system during haptic contact modified by mirror illusion. We believe that looking deeper into the neurophysiological correlations of touch can contribute to a better understanding of its role and increase the effectivity of its application in therapy to its full potential.

The objective of our study is to evaluate and localize changes in the intracerebral source activity via sLORETA imaging during the haptic stimulation of hands, while modifying this contact by a mirror illusion in comparison to a calm state with open eyes using electroencephalography.

## 2. Materials and Methods

### 2.1. Participants

A total of 10 healthy adults, 5 male, 5 female, aged 23–42 years, were included in the study (Table 1). All subjects were right-hand dominated. Severe neurological impairment and vision impairment in medical history were contraindications for inclusion. 

This study was approved by the Charles University FTVS Ethics Committee (255/2018), and all participants submitted their written consent. 

### 2.2. Experimental Design and Procedure

The brain activity was measured using “Wireless EEG Nicolet” device using specialized Electro-Cap with 19 built-in electrodes (Fp1, Fp2, F3, F4, C3, C4, P3, P4, O1, O2, F7, F8, T3, T4, T5, T6, Fz, Cz, Pz) distributed according to the international system 10/20. The sampling frequency was 500 Hz, with a band range of 0.5–70 Hz, and impedance resistance below 10 kΩ [25].

**Figure 1:**
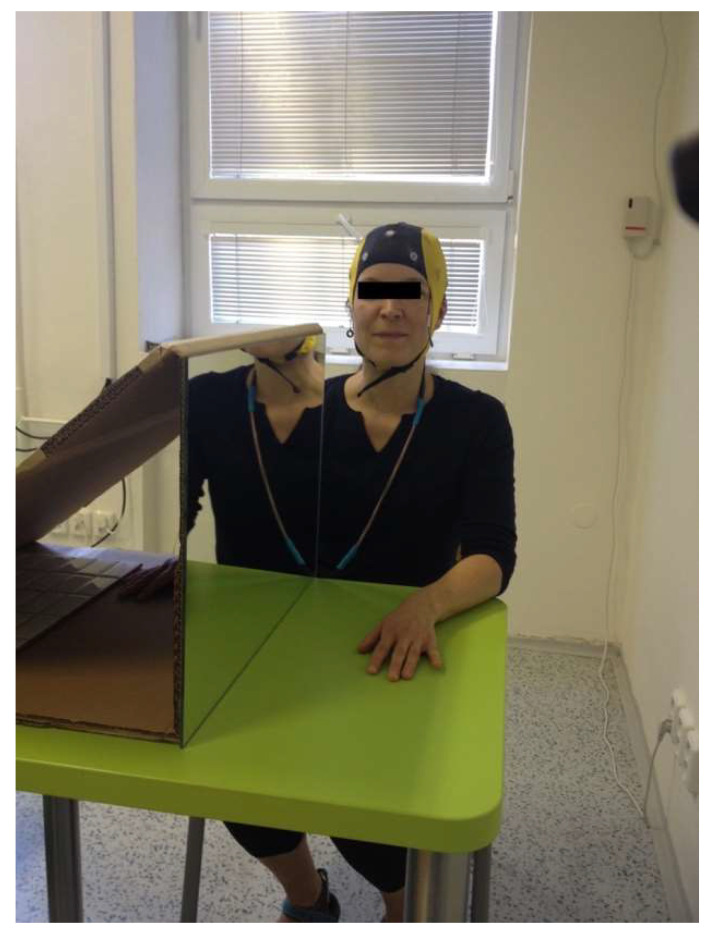
Position of the participant wearing the EEG cap and placing of the mirror during the experiment.

The haptic contact was modified by a mirror sized 55 cm × 40 cm placed sagittally to the tested subject (Figure 1). The mirror was facing a white wall to eliminate object reflections and maintain homogeneity. Tested subjects were not wearing wrist watches nor jewelry [26]. 

The experiment was composed of 5 phases. First, we recorded the brain activity during resting state with open and with closed eyes, each for 5 min. Afterwards, the participants were seated at a table with a mirror reflecting their left hand and forearm and occluding their right hand and forearm (Figure 1). The brain activity was then recorded during 4 modifications of the experiment in duration of 2 min each. The first task contained symmetrical haptic contact on both hands (BOTH), the second modification involved stimulation of the left hand only (LEFT), during the third task, the stimulus was applied on the right hand only (RIGHT) and during the last modification, we did not offer any tactile stimulus (NONE). The order of modifications for each individual was randomized. The recordings were completed in a single trial to avoid burn-out of the mirror illusion. Sources of variation were eliminated to the maximum by conducting the haptic contact by the same researcher in all cases. It contained light stroking by fingertips and palms of hands down the back of the hands and distal forearms of the participants in the same movement pattern. The room was always air-conditioned to the same temperature and the experiment took place in two subsequent days.

Once all the modifications were recorded, the participants were questioned regarding their subjective experience. The survey areas were based on symptoms commonly occurring during mirror therapy [26], which were listed in a prepared questionnaire. However, the participants did not see the list and described their observations freely in their own words. The areas included overall impression, findings related to emotions, vegetative changes, sensations on upper limbs and body scheme shifts. The second part of the survey was designed to gather personal and anamnestic data.

Securing the data set for each participant took approximately 1 h including preparations, EEG recordings and the survey.

In this presented study, sLORETA (standardized low resolution brain electromagnetic tomography) program was used. The program enables one to localize the source brain activity based on standardized current density imaging [27,28]. The method is based on calculating the current density from the entire brain volume. The current density distribution is calculated in voxels—spatial units. The sLORETA program determines current densities in a total of 6430 voxels with a space resolution of 5 mm. For localizing the voxels within the grey matter, sLORETA uses Talairach brain atlas and probabilistic brain atlas. The outputs are 3D brain models highlighting the current distribution of the neural activity [29,30,31].

### 2.3. Data Analysis

The EEG recording resulted in 60 sequences of 2 min (resp. 5 min) each. The segmentation of the data was performed visually by a certified electroencephalographist. Using the NeuroGuide program, 30 s concatenated non-artifact segments were selected and exported into the sLORETA program [25]. The selection was made based on the frequency and amplitude characteristics with respect to the area of occurrence. The recording, as well as the conversion of the data, were also conducted by the experienced electroencephalographist. The conversion was conducted by calculating the reciprocal spectrum in a parametrical model for multichannel EEG. The calculation was made for all the band ranges (delta 0.5–4 Hz, theta 4–8 Hz, alpha 8–10 Hz, alpha2 10–12 Hz, beta-1 13–18 Hz, beta-2 18–21 Hz, beta-3 21–30 Hz and gamma (>30 Hz)) [32,33]. Subsequently, the data were converted into .slor files which enable viewing in the Talairach cortical atlas [34]. A transformation matrix secured by the conversion of the electrode coordinates from the native EEG was used for the data transfer [29]. 

The statistical analysis of the data was also executed using sLORETA. Four pair groups of data were mutually compared in the statistical module as follows:

1. Tactile stimulation of both hands modified by mirror illusion vs. calm state with open eyes (BOTH vs. OE).
2. Tactile stimulation of the left hand modified by mirror illusion vs. calm state with open eyes (LEFT vs. OE).
3. Tactile stimulation of the right hand modified by mirror illusion vs. calm state with open eyes (RIGHT vs. OE).
4. A modification with mirror illusion but no stimuli applied vs. calm state with open eyes (NONE vs. OE).

The changes in the electrical brain activity of all 10 subjects were compared for each pair group. To evaluate statistically significant changes in the source activity, a pairwise *t*-test with logarithmical transformation of the data with the aliasing parameter of 0.5 with permutation method using 5000 randomizations was used. The significance level was *p* ≤ 0.05.

The data were subsequently viewed in the sLORETA Viewer module, which shows the statistically significant changes in the current density in single Brodmann areas for each frequency band. The outputs are 2D images of the brain in single plains, 3D spherical brain model and a text file listing the relevant Brodmann areas.

## 3. Results

### 3.1. Results Summary

The results are summarized in Table 2. The table contains results from all four modifications, describes characteristics of frequency bands and lists descriptions of Brodmann areas relevant to our study. Detailed results of each modification are described in the following Section 3.2, Section 3.3, Section 3.4 and Section 3.5. Section 3.6 contains results of the survey on subjective findings of the participants.

### 3.2. Comparison of the Source Brain Activity during Tactile Stimulation of Both Hands Modified by Mirror Illusion vs. Calm State with Open Eyes (BOTH vs. OE)

A statistically significant difference in the source brain activity was found within the frequency bands beta-2 and beta-3 at the significance level of *p* ≤ 0.05.

**Figure 2:**
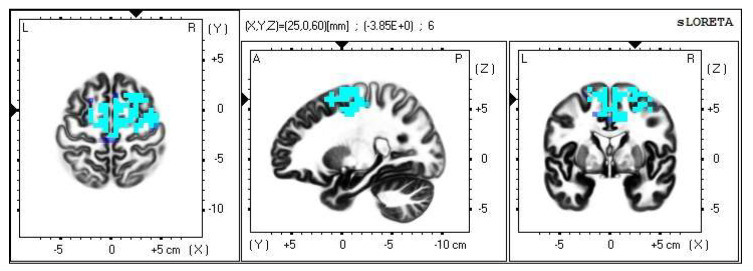
2D image of the statistically significant changes in the current density during the comparison of tactile stimulation of both hands modified by mirror illusion vs. calm state with open eyes at the significance level of *p* ≤ 0.05 in the frequency band beta-2 in BA 6. Transversal, sagittal, and frontal plain. Statistically significant voxels are highlighted in color.

**Figure 3:**
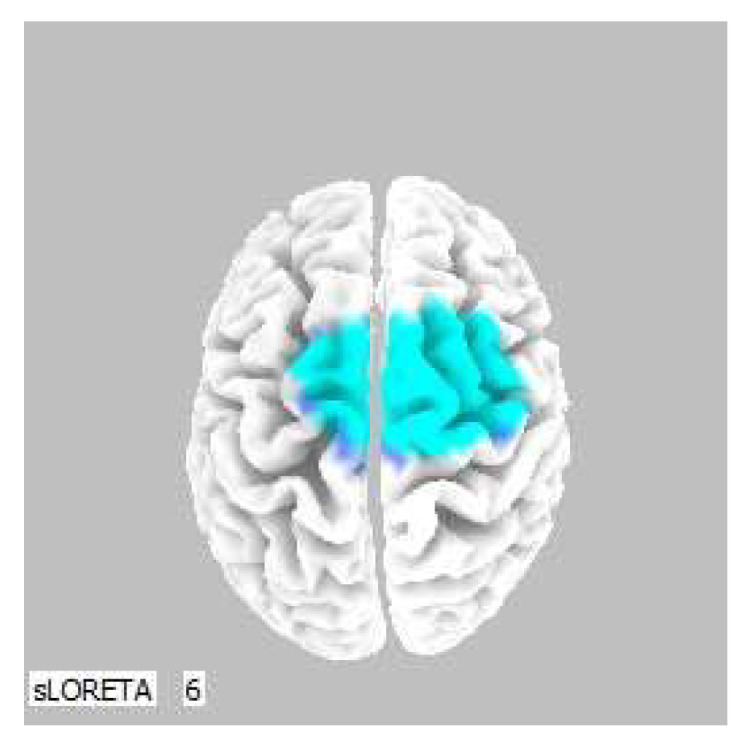
3D image of the statistically significant changes in the current density during the comparison of tactile stimulation of both hands modified by mirror illusion vs. calm state with open eyes at the significance level of *p* ≤ 0.05 in the frequency band beta-2 in BA 6. A view from above. Statistically significant voxels are highlighted in color.

A significant increase in the current density was detected in the frequency band beta-2 in the frontal lobe (BA 6), more frequently in the right brain hemisphere (Figure 2 and Figure 3).

**Figure 4:**
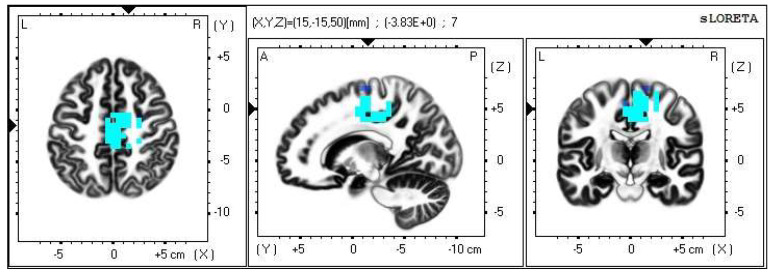
2D image of the statistically significant changes in the current density during the comparison of tactile stimulation of both hands modified by mirror illusion vs. calm state with open eyes at the significance level of *p* ≤ 0.05 in the frequency band beta-3 in BA 6, BA 31 and BA 24. Transversal, sagittal and frontal plain. Statistically significant voxels are highlighted in color.

**Figure 5:**
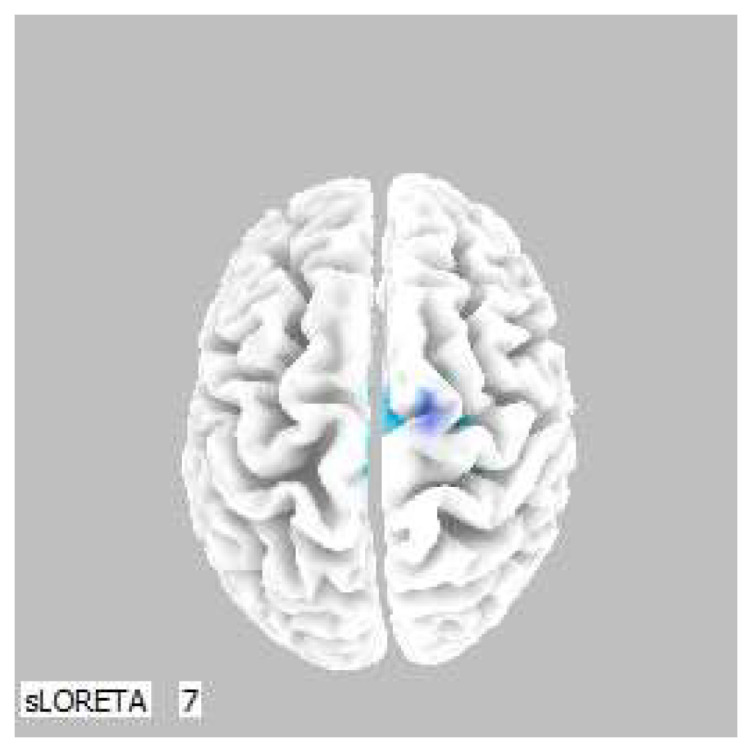
3D image of the statistically significant changes in the current density during the comparison of tactile stimulation of both hands modified by mirror illusion vs. calm state with open eyes at the significance level of *p* ≤ 0.05 in the frequency band beta-3 in BA 6, BA 31 and BA 24. View from above. Statistically significant voxels are highlighted in color.

Within the frequency band beta-3, the significant increase in the current density was detected in the frontal lobe and the limbic area (BA 6, BA 31 and BA 24), also more frequently in the right brain hemisphere (Figure 4 and Figure 5).

### 3.3. Comparison of the Tactile Stimulation of the Left Hand Modified by Mirror Illusion vs. Calm State with Open Eyes (LEFT vs. OE)

A statistically significant difference in the source brain activity was found within the frequency band beta-2 at the significance level of *p* ≤ 0.05.

**Figure 6:**
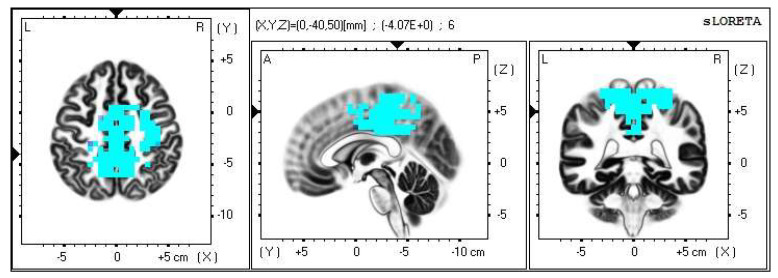
2D image of the statistically significant changes in the current density during the comparison of tactile stimulation of left hand modified by mirror illusion vs. calm state with open eyes at the significance level of *p* ≤ 0.05 in the frequency band beta-2 in BA 31, BA 5 and BA 7. Transversal, sagittal and frontal plain. Statistically significant voxels are highlighted in color.

**Figure 7:**
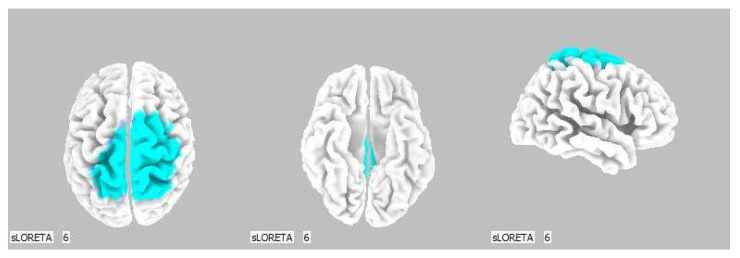
3D image of the statistically significant changes in the current density during the comparison of tactile stimulation of left hand modified by mirror illusion vs. calm state with open eyes at the significance level of *p* ≤ 0.05 in the frequency band beta-2 in BA 31, BA 5 and BA 7. View from above, below and right. Statistically significant voxels are highlighted in color.

A significant increase in the current density was detected in the frontal lobe (BA 31) and in the parietal lobe (BA 5 and BA 7), more frequently in the right brain hemisphere (Figure 6 and Figure 7).

### 3.4. Comparison of the Tactile Stimulation of the Right Hand Modified by Mirror Illusion vs. Calm State with Open Eyes (RIGHT vs. OE)

A statistically significant difference in the source brain activity was found within the frequency band delta at the significance level of *p* ≤ 0.05.

**Figure 8:**
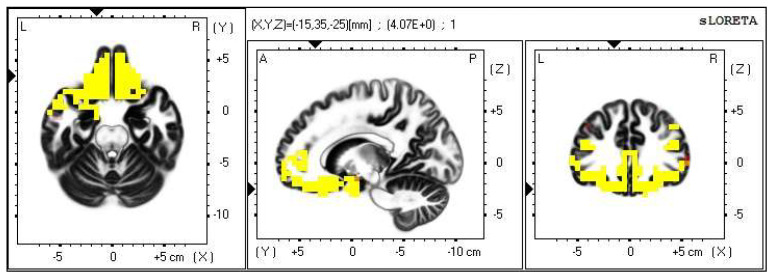
2D image of the statistically significant changes in the current density during the comparison of tactile stimulation of right hand modified by mirror illusion vs. calm state with open eyes at the significance level of *p* ≤ 0.05 in the frequency band delta in BA 11 and BA 47. Transversal, sagittal and frontal plain. Statistically significant voxels are highlighted in color.

**Figure 9:**
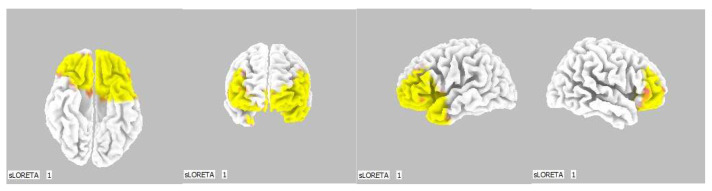
3D image of the statistically significant changes in the current density during the comparison of tactile stimulation of right hand modified by mirror illusion vs. calm state with open eyes at the significance level of *p* ≤ 0.05 in the frequency band delta in BA 11 and BA 47. View from below, front, left and right. Statistically significant voxels are highlighted in color.

A significant increase in the current density was detected in the frontal lobe (BA 11 and BA 47), more frequently in the left brain hemisphere (Figure 8 and Figure 9).

### 3.5. Comparison of the Source Brain Activity during a Modification with Mirror Illusion but No Stimuli Applied vs. Calm State with Open Eyes (NONE vs. OE)

A statistically significant difference in the source brain activity was found within the frequency bands delta and beta-3 at the significance level of *p* ≤ 0.05.

**Figure 10:**
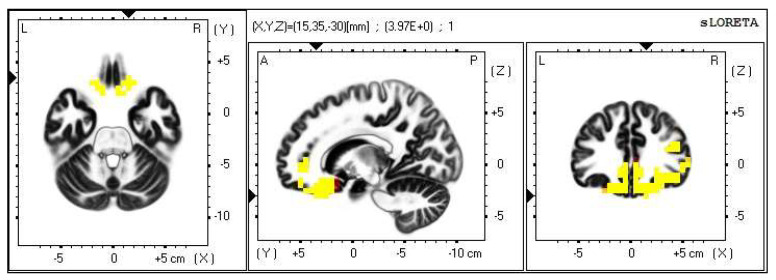
2D image of the statistically significant changes in the current density during the comparison of a modification with mirror illusion but no stimuli applied vs. calm state with open eyes at the significance level of *p* ≤ 0.05 in the frequency band delta in BA 11 and BA 47. Transversal, sagittal and frontal plain. Statistically significant voxels are highlighted in color.

**Figure 11:**
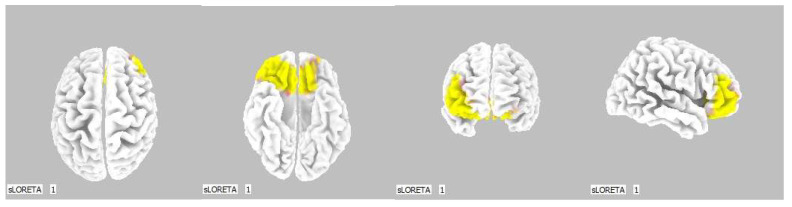
3D image of the statistically significant changes in the current density during the comparison of a modification with mirror illusion but no stimuli applied vs. calm state with open eyes at the significance level of *p* ≤ 0.05 in the frequency band delta in BA 11 and BA 47. View from above, below, front and right. Statistically significant voxels are highlighted in color.

A significant increase in the current density was detected in the frequency band delta in the frontal lobe (BA 11 and BA 47), more frequently in the right brain hemisphere (Figure 10 and Figure 11).

**Figure 12:**
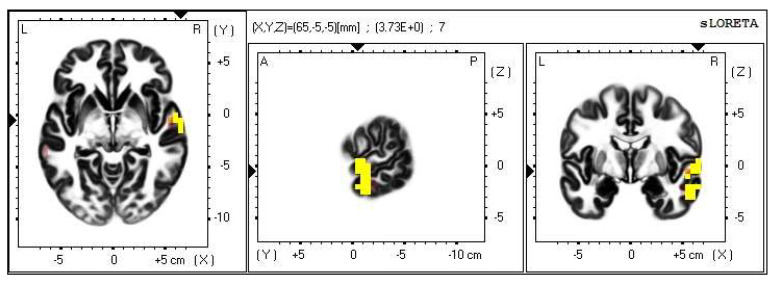
2D image of the statistically significant changes in the current density during the comparison of a modification with mirror illusion but no stimuli applied vs. calm state with open eyes at the significance level of *p* ≤ 0.05 in the frequency band beta-3 in BA 21 and BA 20. Transversal, sagittal and frontal plain. Statistically significant voxels are highlighted in color.

**Figure 13:**
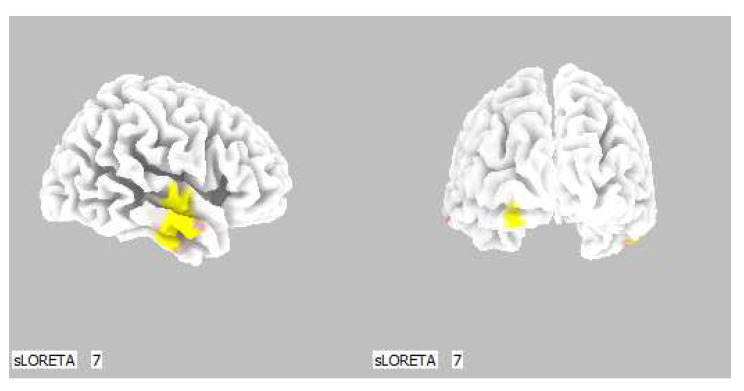
3D image of the statistically significant changes in the current density during the comparison of a modification with mirror illusion but no stimuli applied vs. calm state with open eyes at the significance level of *p* ≤ 0.05 in the frequency band beta-3 in BA 21, BA 20 and BA 18. View from the right and back. Statistically significant voxels are highlighted in color.

Within the frequency band beta-3, the significant increase in the current density was detected in the temporal lobe (BA 21, and BA 20), also more frequently in the right brain hemisphere. Additionally, a significant increase in the current density was localized in the occipital lobe (BA 18) in the left brain hemisphere (Figure 12 and Figure 13).

### 3.6. Results of the Survey Collecting Subjective Evaluation of the Participants

A complementary part of the study was a survey. Its purpose was to gather personal and anamnestic data from all tested subjects and to record their subjective observations. The participants were guided by the researcher to describe their overall impression, findings related to emotions, vegetative changes, sensations on upper limbs and body scheme shifts (Table 3). Answering each question was voluntary. The modification with the most comments was the second one (LEFT vs. OE), probably because it contained the sensory conflict. This modification also had the most recorded cases of paresthesia, which can simply be described as a phantom touch sensation. Subjectively, the experiment was considered as overall pleasant. Interestingly, the least favorite part was again the LEFT vs. OE task.

## 4. Discussion

The objective of our study is to evaluate and localize changes in the intracerebral source activity during the haptic stimulation of hands while modifying this contact by a mirror illusion. 

The statistically significant difference in source brain activity occurred during all four modifications of our experiment, resulting in the activation of 10 different Brodmann areas in three different frequency bands varying by the modification of the provided stimuli. 

Throughout our entire experiment, the activated areas were often the ones involved in visual perception and processes of visual and motor integration. The systems processing the tactile and visual stimuli cooperate and influence one another. Touch can increase the brain’s sensitivity toward other modalities and enable stimuli to pass the threshold of awareness. The interaction between visual and tactile inputs takes place in the beginning of the cortical processing, and the integration of various modality stimuli does not have to take place on the cognitive level [20]. 

Interestingly, in the two modifications of our experiment during which the participants could actually see the performed haptic contact (BOTH vs. OE and LEFT vs. OE), the areas known for containing mirror neurons were activated (BAs 6, 24, 5 and 7). 

Mirror neurons are specific cells that discharge during purpose-motivated movement, but also while such action is only observed in another individual. These cells were firstly described in the ventral premotor cortex (area F5) in Macaque monkeys in 1992 [44]. The following research proved their existence in other parts of the brain in monkeys and other primates, and later also in humans [9,10].

Besides the primary motor cortex, which plays, of course, the central part in executing voluntary movement, the mirror neurons can be found in other areas related to motor functions of the hand, foot and mouth, including the Broca’s speech center and in areas related to visceral motor functions related to emotions [14,17,45]. 

The mirror neuron theory is based on specific backgrounds which explain the connection between function and purpose understanding in a goal-directed movement. Most importantly, the mirror neuron activity is motoric in its nature. This means that the motor system is not only responsible for movement execution, but it also takes part in the cognitive processes of understanding side to side with the abstract and symbolic brain functions [46]. 

During the first modification of our experiment (**BOTH vs. OE**), a statistically significant difference in the source brain activity was found within the frequency bands beta-2 and beta-3 at the significance level of *p* ≤ 0.05. 

The most frequent increase in the source brain activity was detected in the Brodmann area (BA) 6 within the beta-2 band and in BAs 6, 31 and 24 within the beta-3 band, more so in the right brain hemisphere.

Brodmann area 6 is part of the premotor cortex of the frontal lobe. It lies in front of the primary motor area, close to the Sylvian fissure. It is probably the largest Brodmann area and, being such, it relates to a vast scale of functions. On the background of our experiment, the functions related to motor processes are the most relevant. These include motor planning, motor learning and motor imaging. This brain region is also involved in sensory guiding during movement, fluent linking of single movement components and coordination of contralateral limbs. This area also functionally belongs with the mirror neuron circuits and activates during activity observing and activity imaging [39,40].

Besides the functions connected to motor skills, the BA 6 is closely connected to specific attention and awareness tasks and plays a role in memory storing processes. It is also involved in the speech processing, new task solving, self-control, emotion processing and self-reflection during decision making [39,40].

The Brodmann areas 24 and 31 are located in the cingulate gyrus. Because this structure is part of the limbic system, it is strongly related to emotions.

The increased activity in the cingulate gyrus in the beta band can indicate increased synchronization of sensory and motor brain regions. Thanks to its location, the cingulate gyrus is anatomically and functionally connected with many other brain regions and is involved in numerous neural circuits. It takes part not only in motor tasks, but also more importantly in the processes of affective and cognitive functions [47]. 

The BA 24 lies within the anterior part of the cingulate gyrus. Regarding the motor functions, it takes part in motor planning and imaging and reacts to vestibular and visual motor inputs. When it comes to somatosensory functions, the BA 24 plays a role in pain stimuli processing. The BA 24 is also involved in the mirror neuron system; specifically, its neurons fire in the processes of distinguishing oneself from others during social interactions.

The Brodmann area 31 is located within the posterior cingulate gyrus. Opposed to the anterior section, this area does not have such a big part in motor processing. However, it is involved in the processes of complex motor skill learning. The BA 31 is commonly being put in relations with speech, emotions and memory. It also comes to action in processing complex visual stimuli, which correlates with the conditions of our experiment [39,40].

The posterior cingulate gyrus is involved in functions such as evaluative judgment, precaution decision making, fear conditioning, distinguishing oneself from others and more.

Based on the characteristics of the activated areas related to awareness, learning and imaging, but also to emotions, which are essential for learning and memory storing processes, we believe this particular situation was clearly a new experience for the tested subjects, regardless of the randomization. This task was the only one offering a symmetrical haptic contact correlating with the visual input, which could support the activation of limb coordination functions [39,40].

Opposed to the above, the **LEFT vs. OE** modification meant an asymmetrical stimulation while the modified touch was observed by the subjects. A statistically significant difference in the source brain activity was found within the frequency band beta-2 at the significance level of *p* ≤ 0.05.

A significant increase in the current density was detected in the frontal lobe (BA 31) and in the parietal lobe (BA 5 and BA 7).

The Brodmann areas 5 and 7 are situated in the parietal cortex representing the secondary somatosensory center. This makes them essential for somatosensory input processing. Their functions include visual motor attention [39,40].

The activation of the BA 5 and BA 7 was detected in both brain hemispheres, but more so in the right one, which indicates activated spatial perception including the sensitivity toward personal space. We offered the stimulus on the correlating limb—the left hand—but it could also indicate that the conditions held higher demands on the spatial orientation, which resulted in the activation of the contralateral sensory regions in the left hemisphere as well.

The cognitive and sensory conflict delivered by the mirror illusion can activate brain areas which could not be targeted by control conditions. This effect is more significant in unilateral or asymmetrical tasks. The brain regions directly activated by mirror illusion include the posterior cingulate gyrus and the superior parietal lobe in both brain hemispheres, which includes the contralateral secondary somatosensory cortex (BA 5 and BA 7) [48]. 

This modification placed increased demands on the localization abilities due to the mirror illusion. Regarding the tactile perception, the processes of localization take place in the superior somatosensory center, while the inferior parts (BA 40 and BA 39) are involved in the processes of object identification [39,40]. 

The BA 5 and BA 7 also take part in the circuits of imitating and motor learning [39,40]. These functions are related to the mirror neuron activity, and given the characteristics of our study, we believe their involvement is logical. The theory of the tactile mirror system can be supported by the fact that the somatosensory cortex in the contralateral hemisphere was activated by the sole touch observation in the mirror.

Amongst other functions, the BA 31 in the posterior cingulate gyrus takes part in processing demanding visual inputs and distinguishing oneself from others [39,40]. The activation of this area could be caused by the fact that the conditions were highly demanding on the ability to orientate within the given situation. Based on the subjective experience, this modification evoked the most controversies. We also noted several vegetative reactions (Table 3) which could correlate with the activation of the BA 31 being related to the affective processes linked to precaution and fear [39,40].

In addition to the above, two probands also noted a change in the body scheme orientation and, interestingly, six cases of paresthesia occurred during this task. This included phantom touch sensation, prickling, burning, twitching and tingling on the contralateral (right) limb. This phenomenon is known as mirror synesthesia and relates to the hyperactivity of the tactile mirror system [1,23]. 

Bolognini et al. (2014) conducted a study focusing specifically on this phenomenon. The authors intended to provoke mirror synesthesia using transcranial magnetic stimulation in healthy individuals observing touch. Simultaneously, they aimed to indicate which brain regions cause the synesthesia via their interaction. The results of their study suggest that the primary somatosensory area S1 is in a close functional linkage with the posterior parietal cortex (BA 5 and BA 7) and the premotor area (BA 6). The bimodal neurons in these areas react to visual and tactile stimulation and are activated specifically while observing touch [1]. During the LEFT vs. OE part of our experiment, this correlation also occurred.

The tactile mirror system and the level of its excitability is rather individual, and to a certain extent, depends on cognitive abilities and affective empathy. The mirror synesthesia occurs in about 1,6% of individuals and is caused by the increased activation of neural networks between the frontal and parietal areas, which are, amongst other functions, involved in the processes of distinguishing oneself from others [23].

The third modification of our experiment (**RIGHT vs. OE**) showed an increase in the source brain activity within the delta frequency band at the significance level of *p* ≤ 0.05. This occurred during modifications, which were probably perceived as the most unclear, and thus demanded increased attention and activation of higher cognitive functions and intelligence capacity [29,41,42,43].

A significant increase in the current density within the delta band was detected in the frontal lobe (BA 11 and BA 47), more so in the left brain hemisphere.

The results of this particular modification suggest that the tested subjects were attempting to localize the occluded tactile stimulus which did not correlate with the visual input. Subjectively, some tested individuals rated this modification as peculiar, and they mentioned affective feelings of confusion. Several vegetative reactions also occurred. This part of the experiment caused the most body scheme orientation shifts. Based on the subjective evaluation, but more importantly, on the results retrieved from the EEG recordings and statistical processing, we believe that this part of the experiment was the most confusing both cognitively and sensory-wise (Table 3).

This part of the experiment obtained an asymmetrical tactile stimulus, which additionally took place behind the mirror, and was thus veiled by a false visual input. 

The Brodmann areas 11 and 47 lie in the frontal lobe, in the gyri inferioris and rectus. Their functions are primarily related to speech, semantic processes and language understanding, but they are also connected to non-verbal communication. These two BAs also take part in inhibiting negative emotions, in the attribution of intentions to others and intuition. It seems logical that such an unclear situation raised the demands on the activation of the areas involved in the above [39,40].

The increased activity occurred in both brain hemispheres, more frequently in the left one, which is in accordance with the fact that the tactile stimulus was offered on the right hand. The limb was, however, occluded by the mirror; therefore, such input probably caused the cognitive and sensory conflict mentioned earlier, resulting in the contralateral brain activity as well.

As described in the introduction of this article, touch is one of the basic communication tools, and being such, it plays an essential role in interaction between individuals. The conditions of our experiment led to the activation of several areas involved in speech and language understanding, non-verbal communication, and semantic processing; those related to the attribution of intentions to others, and also areas involved in the mirror neuron system. The mirror neurons fire not only during motor imitating processes, but also bear functions related to communication, language and social behavior [49]. This means that the conditions of our experiment might have caused both conscious and subconscious attempts to interpret the given situation as a form of communication. 

When it comes to the last modification (**NONE vs. OE**), a statistically significant difference in the source brain activity was found within the frequency bands delta and beta-3 at the significance level of *p* ≤ 0.05.

A significant increase in the current density was detected in the frequency band delta in the frontal lobe (BA 11 and BA 47), more frequently in the right brain hemisphere.

Within the frequency band beta-3, the significant increase in the current density was detected in the temporal lobe (BA 21 and BA 20), also more so in the right brain hemisphere.

In addition, a significant increase in the current density was localized in the occipital lobe (BA 18) in the left hemisphere.

The relevant functions of the BA 11 and BA 47 are described above.

The BAs 20 and 21 are involved in the processes of visual integration, and together with the BA 11 and BA 47, they are involved in the processes of attribution of intentions to others [39,40].

The BA 21 is found in the medial part of the temporal lobe. Its activity is most importantly related to speech understanding. Other functions include the attribution of intentions to others; nevertheless, the BA 21 usually cooperates with the other cortex regions on such processes.

The BA 20 lies within the inferior temporal lobe, gyri fusiformis and parahippocampus. It is activated during complex visual stimuli processing and recognition processes. According to several studies, the BA 20 is included in the Wernicke’s speech center. Particularly in the left brain hemisphere, the BA 20 takes part in speech understanding. Being a part of the gyrus fusiformis, it also plays a role in the processes of including visual elements into a sensory complex and also activates during visual fixation. Similarly to the BA 21, the BA 20 also participates in attributing intentions to others [39,40].

The Brodmann area 18 lies in the medial part of the occipital lobe. Most importantly, it represents the secondary visual cortex; thus, its main function remains the integration of visual inputs. The modalities processed in the BA 18 include light intensity detection; movement structure and pattern detection; determination of the gestures of single fingers; attention toward colors and shapes and other functions. However, it also takes part in the processes related to memory and language understanding. In the right hemisphere, the BA 18 engages in the processes of emotions and attention related to visual integration. In the left hemisphere, the processes of visual mental integration take place [39,40].

If this modification of our experiment should be described in one word, it would have to be the word “expectation”. In both activated frequency bands, the results have a common denominator—the evaluation of the intentions of another person. We believe that the participants experienced expectation very strongly. This result, however, could be boosted by the fact that this modification was never carried out at first, despite the randomization. We can assume that by having the experience with the other modifications, the probands anticipated some form of haptic contact from the researcher. Its absence probably led to an increased activation of the secondary visual cortex which integrates visual inputs, as discussed above. 

The participants of our experiment were healthy adults; thus, we cannot claim that the conditions of our study would have the same influence on individuals with health issues. That would have to be a subject of follow-up research. Nevertheless, we believe our findings may have potential for therapy.

The principles of mirror visual feedback and backgrounds of mirror neuron theory are already being commonly used in healthcare for treating motor impairments as well as sensory issues including pain [11,14]. One of the greatest benefits is the fact that the relevant brain regions can be trained without the necessity to execute the actual movement [7,14,50].

Amongst other findings, we were able to activate the regions synchronizing motor and sensory functions, which could potentially facilitate the feedback mechanisms of the CNS [13,50].

The areas responsible for motor planning and imaging were also activated, which is essential for building a base for the recovery of motor functions [14,16] and facilitating neuroplasticity [51,52]. 

We also recorded the activity in the brain regions related to emotions and memory processes, which provide a crucial baseline for learning new motor skills and rewriting motor programs [47]. 

Lastly, we believe our findings could be beneficial for investigating the possibilities of addressing sensory aphasia, since several areas involved in speech understanding were also activated.

Despite our significant results, we are aware that our study bears its limitations. The group of tested subjects was rather small and homogeneous. In our experiment, we investigated the neurophysiological context of interpersonal haptic contact; therefore, we could not completely eliminate the variations in all qualities of the provided touch such as body temperature, pressure and stroking pace. We did not conduct measurements in the control conditions without using the mirror illusion, but it was not part of our objectives. 

## 5. Conclusions

The presented electroencephalographic analysis suggests that the summation of stimuli secured by interpersonal haptic contact modified by the mirror illusion can result in activating brain areas integrating motor, sensory and cognitive functions, and further areas related to communication and understanding processes including the mirror neuron system. We believe these findings may have therapeutical potential in the areas of curing motor impairments, pain management and sensory deficits.

## Figures and Tables

**Table 1:** Basic characteristics of the participants.

Participants	Gender	Age (Years)	Height (cm)	Body Mass (kg)	BMI (kg/m^2^)
10	50% M, 50% F	34.4 ± 6.422	171.3 ± 7.363	67.7 ± 12.256	22.98 ± 3.344

**Table 2:** Statistically significant changes in the source activity for each pair group at the significance level of *p* ≤ 0.05; the frequency bands; activated Brodmann areas (BAs) and their functions relevant to our study.

Pair Group	Frequency Band	Frequency Band Characteristics	Source Activity Localization	BA	Relevant BA Functions
BOTH vs. OE	beta-2	Attention, focus, awareness, tactile stimulation [29,35,36,37,38].	Frontal lobe—Premotor cortex	6	Motor planning, learning and imaging; movement guidance and fluency; coordination; mirror neurons; awareness; learning; memory; emotions; speech; new experiences [39,40].

	beta-3		Cingulate gyrus—Anterior part	24	Motor planning, imaging; visual attention; mirror neurons; memory; emotions; self-reflection; new experiences [39,40].
			Cingulate gyrus—Posterior part	31	Learning complex motor skills; motor visual stimuli; memory; emotions; precaution; fear; speech; self-reflection [39,40].
LEFT vs. OE					
	beta-2		Superior parietal lobe—Secondary sensorimotor cortex	5, 7	Somatosensory fcs; visuomotor attention; spatial perception and memory; object localization; mirror neurons; self-reflection during decision making [39,40].
RIGHT vs. OE	delta	Memory, intelligence, decision making, stimuli detection, anticipation, spatial orientation [29,41,42,43].	Frontal lobe—gyrus rectus. Inferior frontal gyrus	11, 47	Speech, semantics; smell; hearing; decision making; negative emotion inhibition; attribution of intentions to others; intuition [39,40].


NONE vs. OE	beta-3	Attention, focus, awareness, tactile stimulation [29,35,36,37,38].	Temporal lobe—inferior and medial part	20, 21	Speech; complex processing of visual inputs; recognition; attribution of intention to others, motion observation [39,40].
			Occipital lobe—secondary visual area	18	Visual integration; speech; memory; emotions; visual imaging [39,40].

vs., versus; OE, open eyes; BA, Brodmann area; fcs, functions.

**Table 3:** Subjective evaluation of the participants. The figures represent number of comments on the given modification in the presented manner. Total number of comments is in bold.

	BOTH vs. OE	LEFT vs. OE	RIGHT vs. OE	NONE vs. OE	Comments Total
**Comments total**	**15**	**27**	**22**	**13**	
**Overall evaluation**					
Pleasant	4	3	2	1	**10**
Unpleasant		4	1		**5**
Neutral	2			2	**4**
Peculiar			2		**2**
Pleasant yet peculiar	1	2	1	1	**5**
**Emotional reaction**					
Confusion		1	2		**3**
Insecurity	1			1	**2**
Nervousness	1	1	1	1	**4**
Expectation				2	**2**
**Vegetative reaction**					
Higher pulse rate	1	1	2	1	**5**
Perspiration		1	1		**2**
Cold		1	1		**2**
**Sensations on upper limbs/** **body scheme shifts**					
Temperature difference		1			**1**
* Paresthesia		6	1		**7**
Urge to move limb		2	1	1	**4**
Touch outside of limb	1	1			**2**
“Alienated” limb	1	1	1		**3**
3 limbs—self			2		**2**
3 limbs—researcher			1		**1**
Hypesthesia	1		1		**2**
Touch expectation				1	**1**
**Other**					
Urge to close eyes	1	1	1	1	**4**
Illusion burn-out	1	1	1	1	**4**

* Paresthesia includes sensations of phantom touch, prickling, burning, twitching, tingling, etc.; vs., versus; OE, open eyes.

## Data Availability

The data sets generated for this study are available upon reasonable request to the corresponding authors.
